# Accessibility and partner number of protein residues, their relationship and a webserver, ContPlot for their display

**DOI:** 10.1186/1471-2105-10-103

**Published:** 2009-04-08

**Authors:** Arumay Pal, Ranjit Prasad Bahadur, Partha Sarathi Ray, Pinak Chakrabarti

**Affiliations:** 1 Department of Biochemistry, Bose Institute, P-1/12 CIT Scheme VIIM, Kolkata 700 054, India; 2 Bioinformatics Centre, Bose Institute, P-1/12 CIT Scheme VIIM, Kolkata 700 054, India

## Abstract

**Background:**

Depending on chemical features residues have preferred locations – interior or exterior – in protein structures, which also determine how many other residues are found around them. The close packing of residues is the hallmark of protein interior and protein-protein interaction sites.

**Results:**

The average values of accessible surface area (ASA) and partner number (PN, the number of other residues within a distance of 4.5 Å from any atom of a given residue) of different residues have been determined and a webserver, ContPlot has been designed to display these values (relative to the average values) along the protein sequence. This would be useful to visually identify residues that are densely packed, or those involved in protein-protein interactions. The skewness observed in the distribution of PNs is indicative of the hydrophobic or hydrophilic nature of the residue. The variation of ASA with PN can be analytically expressed in terms of a cubic equation. These equations (one for each residue) can be used to estimate the ASA of a polypeptide chain using the PNs of the individual residues in the structure.

**Conclusion:**

The atom-based PNs (obtained by counting surrounding atoms) are highly correlated to the residue-based PN, indicating that the latter can adequately capture the atomic details of packing. The average values of ASA and PN associated with each residue should be useful in protein structure prediction or fold-recognition algorithm. ContPlot would provide a handy tool to assess the importance of a residue in the protein structure or interaction site.

## Background

Hydrophobic interactions have long been hypothesized to play a dominant role in organizing and stabilizing the architecture of proteins [1,2]. This causes the non-polar side chains to segregate into the interior of globular proteins constituting the core, which is compact, densely packed and can be viewed as being solid-like [3]. The charged and polar side-chains remain exposed to the solvent. Thus the pattern of hydrophobic and polar residues may constitute the binary code responsible for the fold of a protein [4,5]. The dependence of the degree of burial of a residue on its hydrophobicity [6] would mean that its solvent accessibility and the number of contacts with the surrounding residue would also show distinct pattern. Indeed, it has been shown that when the contact is defined in terms of the number of atoms (i.e., partner number) surrounding a given residue the above two parameters are also related [7]. Parameters such as these obtained from a database of known protein structures constitute the knowledge base for deriving scoring functions or statistical potentials used in protein fold recognition, usually by threading, concerned with protein-structure prediction for cases in which a target sequence does not have unambiguous sequence homology to any known structure [8-12]. These knowledge-based potential functions could be at the residue level or atom-based [13-15]. As the earlier work [7] defined the partner number (PN) in terms of atoms, the present study examines it in terms of residues and finds the relationship with the solvent accessible surface area (ASA). The analytical expression can be used to calculate the ASA corresponding to any observed PN, which can then be compared to the observed ASA to assess the importance of any residue at the binding site [16]. Finally, a webserver is described that scans a protein sequence in a structure to indicate the residues for which the two parameters deviated significantly from the corresponding average value for the same residue.

## Results and discussion

### Partner number of residues

**Figure 1:**
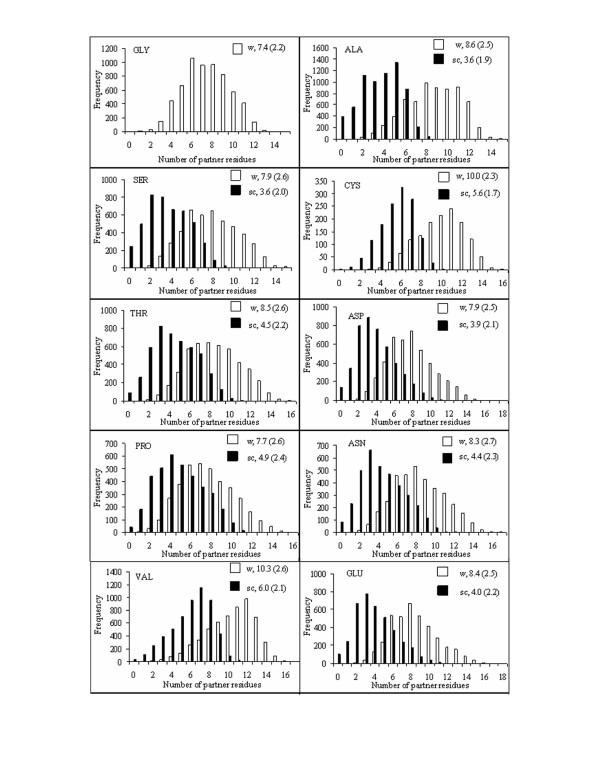
**Histogram showing the distribution of the number of partner residues in contact with a particular amino acid residue in proteins**. The bars corresponding to the side chain (sc) and the whole residue (w) appear grouped towards the left and the right sides of the plot, respectively, and the average value (with standard deviation) of partner-residue in each case is shown. The residues are arranged according to their volume [33]. Only ten residues are shown.

**Figure 2:**
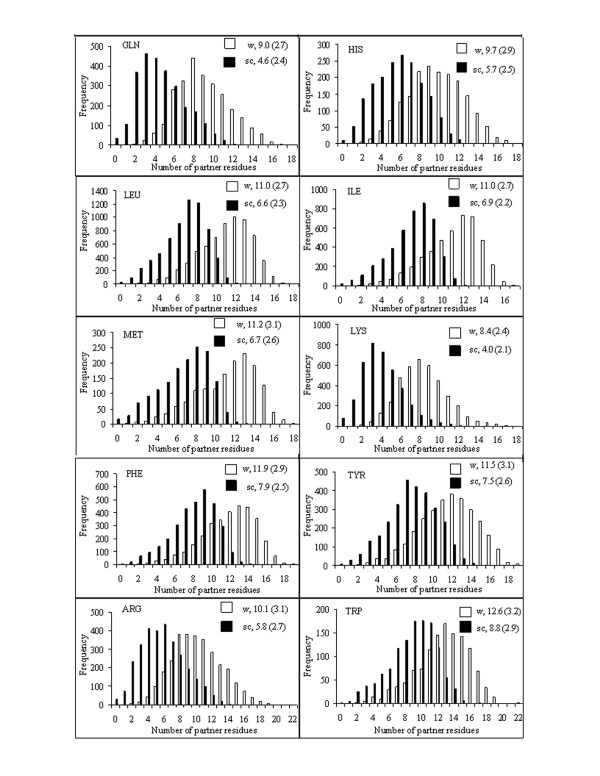
**Same as in Figure 1, but for the remaining ten residues**.

The number of partner residues gives the quantitative measure of the packing of residues in the protein structures. Figure 1 and 2 show the distributions of the number of partner groups around the whole residue of a particular type or just its side chain, and follows the trend observed when this number was calculated counting the individual atoms around the central group [7]. For example, the largest residue Trp has the highest average partner number (12.6 and 8.8 for whole residue and side chain, respectively), whereas the smallest, Gly has the least (7.4). For residues other than Gly the difference in partner numbers for the whole residue and the side chain (i.e., the number interacting with the main-chain atoms) is around 4, which is nearly a third of what was observed while considering the atoms instead of residue as partner [7].

There have been earlier studies analyzing the spatial neighbors of residues in protein structures, though using longer cut-off distances and usually representing residues by a single interaction site located at the C^α ^position [17-20]. The atom-based partner numbers gave good correlation [7] with only the parameters determined by Karlin et al. [19]. However, the atom- and residue-based partner numbers are correlated – the correlation coefficients being 0.95 and 0.94 for the sets determined for the whole residue and the side chain, respectively – indicating that the residue-based calculations can capture the essence of details inherent in the atom-based method. As expected, the hydrophilic residues have lesser partner number compared to the hydrophobic residues of similar volume, for example, Lys vis-à-vis Met. However, the two types can also be distinguished on the basis of the asymmetric (i.e., skewed) nature of the distribution in the partner numbers, especially the ones involving the side chains. Considering Asp and Lys as examples for hydrophilic residues it can be seen that the distributions have positive skewness, with the peaks shifting towards lower partner numbers. In contrast, the distributions have negative skewness (peaks shifting towards higher partner numbers) for hydrophobic residues, such as Leu, Ile and Met.

### Variation of the accessible surface area with the number of partner residues

**Figure 3:**
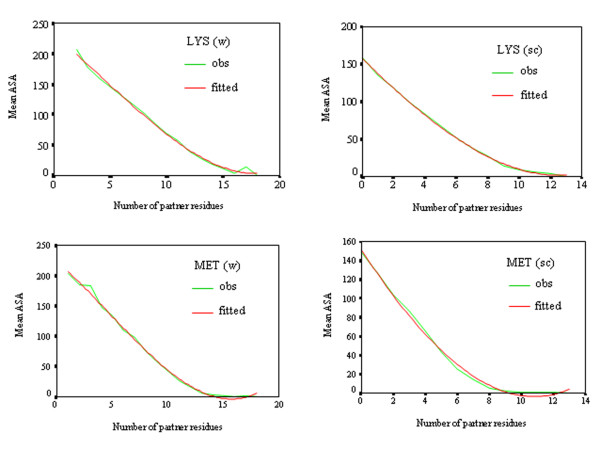
**Variation of the mean accessible surface area (Å^2^) with partner number for Lys and Met, both for the whole residue (w), as well as the side chain (sc)**. The curves corresponding to the best least-squares fit, y = b_0 _+ b_1_x + b_2_x^2 ^+ b_3_x^3 ^(with b_0_, b_1_, b_2 _and b_3 _given in Table 1) are also shown.

Various contact measures, such as the one discussed in the previous section, have been used to evaluate the accuracy of three-dimensional protein models, as also the contact area between residues [21]. To relate the two we have plotted the variation of the mean values of the ASA of the residues (considering either the whole residue or only the side chain) with the partner numbers in Figure 3 for two representative cases (others are given in the Additional file 1, Figure S1). The dependence can be represented by a cubic equation (Table 1). Other curve fitting equations (exponential and quadratic) were also tried, but the R^2 ^values were poorer compared to those obtained from the cubic fitting. For a few residues and especially for the side chain, the fitted plots tend to indicate negative values of ASA at higher partner numbers and it may be better to level off the curves at ASA = 0 at such high values of PN. In the majority of the cases, extrapolation to x = 0 (i.e., no partner) leads to a value which is close to the value for the residue (X) obtained in the fragment Gly-X-Gly in an extended conformation. At this value of x, the calculated ASA is given by the value of b_0_, which should be equivalent to the value of a_1 _in the earlier work where an exponential equation was fitted to relate ASA to the atom-based PN [7]. Indeed, the correlation coefficients between the two sets of values are 0.90 and 0.98 considering the whole residue and the side chain, respectively. Considering the whole residue only three amino acids (Cys, Gln and Leu) have values of b_0 _that differ from those provided by the Gly-X-Gly tripeptide by more than 15%. Considering only the side chain, the variation is in the range 10–15% for Ala, Cys and Thr. The deviation is the maximum for Cys and this is the only residue for which the fitted parameter b_1 _is positive. The discrepancy must be due to the fact that Cys residues with free sulfhydryl group, as well as those involved in disulfide bridges have been considered together.

**Table 1:** Parameters for fitting cubic equation (y = b_0_+ b_1_x + b_2_x^2^+ b_3_x^3^) to relate ASA (y) to the partner number, PN (x) for different residues.

Residue^a^	ASA (Å^2^)	Whole residue					Side chain				
	
	Gly-X-Gly^b^	b_0_	b_1_	b_2_	b_3_	R^2^	b_0_	b_1_	b_2_	b_3_	R^2^
Gly 6245	83.9	84.4 *4.40*	-3.4 *2.31*	-1.1 *0.33*	0.07 *0.01*	0.991					

Ala 6688	116.4 (55.4)	130.5 *6.53*	-9.2 *3.42*	-0.9 *0.49*	0.07 *0.02*	0.990	62.3 *2.86*	-15.9 *3.30*	0.4 *1.00*	0.08 *0.08*	0.989

Ser 4614	125.7 (69.1)	140.0 *3.89*	-12.6 *2.04*	-0.5 *0.29*	0.05 *0.01*	0.997	74.3 *2.14*	-17.6 *1.95*	0.9 *0.47*	0.01 *0.03*	0.994

Cys 1367	141.5 (82.1)	65.6 *12.37*	3.4 *4.79*	-1.5 *0.55*	0.06 *0.02*	0.982	65.6 *2.62*	-11.3 *2.39*	-0.3 *0.57*	0.08 *0.04*	0.990

Thr 4724	148.1 (88.6)	156.7 *4.17*	-10.5 *2.06*	-0.9 *0.28*	0.06 *0.01*	0.997	101.7 *2.65*	-19.1 *1.99*	0.7 *0.40*	0.02 *0.02*	0.994

Asp 4463	155.4 (97.8)	170.9 *4.02*	-15.4 *1.78*	-0.1 *0.22*	0.03 *0.01*	0.997	107.6 *1.34*	-18.2 *1.10*	0.3 *0.24*	0.04 *0.01*	0.999

Pro 3701	144.8 (106.4)	147.2 *4.16*	-6.5 *2.06*	-1.3 *0.28*	0.08 *0.01*	0.997	103.7 *2.70*	-10.8 *2.03*	-0.9 *0.40*	0.09 *0.02*	0.995

Asn 3537	168.9 (109.9)	173.0 *4.49*	-11.8 *2.10*	-0.7 *0.27*	0.05 *0.01*	0.997	116.9 *2.24*	-20.4 *1.55*	0.7 *0.28*	0.01 *0.01*	0.997

Val 5619	162.2 (103.1)	167.8 *7.37*	-15.1 *3.05*	-0.4 *0.36*	0.04 *0.01*	0.995	111.8 *2.51*	-21.6 *2.07*	0.7 *0.45*	0.04 *0.03*	0.996

Glu 3843	187.2 (132.5)	190.8 *4.47*	-9.7 *1.77*	-1.0 *0.20*	0.05 *0.01*	0.999	134.1 *0.79*	-19.4 *0.65*	-0.0 *0.14*	0.06 *0.01*	1.000

Gln 2645	189.2 (129.7)	231.8 *9.56*	-21.9 *3.78*	0.2 *0.42*	0.02 *0.02*	0.995	139.0 *2.27*	-20.5 *1.57*	0.3 *0.29*	0.03 *0.02*	0.998

His 1789	198.5 (141.3)	209.9 *3.31*	-15.9 *1.47*	-0.4 *0.18*	0.04 *0.01*	0.999	147.5 *1.88*	-21.9 *1.41*	0.3 *0.28*	0.04 *0.02*	0.999

Leu 6575	198.0 (141.5)	233.2 *12.42*	-26.7 *4.91*	0.6 *0.55*	0.01 *0.02*	0.990	151.2 *4.90*	-31.7 *2.92*	1.9 *0.48*	-0.03 *0.02*	0.995

Ile 4362	190.0 (130.7)	214.5 *5.24*	-22.6 *2.17*	0.2 *0.26*	0.03 *0.01*	0.998	140.0 *3.39*	-26.2 *2.35*	1.1 *0.43*	0.01 *0.02*	0.995

Met 1533	210.6 (150.4)	223.0 *5.88*	-15.7 *2.61*	-0.8 *0.31*	0.05 *0.01*	0.996	151.7 *3.20*	-26.1 *2.22*	0.8 *0.41*	0.02 *0.02*	0.996

Lys 3861	207.5 (148.0)	231.0 *8.66*	-15.3 *3.42*	-0.4 *0.38*	0.03 *0.01*	0.996	157.6 *1.37*	-20.6 *0.95*	0.3 *0.17*	0.03 *0.01*	0.999

Phe 3225	223.3 (164.2)	210.3 *7.59*	-11.4 *3.20*	-1.0 *0.37*	0.06 *0.01*	0.993	173.5 *3.14*	-27.0 *2.01*	0.8 *0.34*	0.02 *0.02*	0.997

Tyr 2927	238.3 (180.0)	220.1 *8.06*	-10.0 *3.40*	-1.2 *0.39*	0.06 *0.01*	0.992	174.0 *4.67*	-21.7 *2.79*	0.1 *0.44*	0.04 *0.02*	0.993

Arg 3024	249.3 (190.2)	274.3 *5.26*	-21.1 *1.76*	0.1 *0.17*	0.02 *0.01*	0.998	193.6 *2.05*	-26.8 *1.08*	0.9 *0.15*	0.03 *0.01*	0.999

Trp 1230	265.4 (209.6)	265.8 *10.60*	-20.5 *3.25*	-0.1 *0.29*	0.02 *0.01*	0.996	225.6 *2.84*	-34.2 *1.49*	1.5 *0.21*	-0.01 *0.01*	0.998

Standard error for each parameter is given below in italics.

^a^Total number of each residue type in the non redundant dataset of 432 polypeptide chains is provided.

^b^The values for the whole residue and the side chain (in parentheses) for the Gly-based tripeptide are taken from [7].

### Calculation of ASA of a residue (or the whole protein) using the equation involving the partner number

Advances have been made in the prediction of the ASA of amino acid residues in proteins [22]. Wodak and Janin [23] have developed an analytical approximation to ASA, expressed as a function of interatomic distances only. Using the equation derived here relating ASA and partner-number, it is possible to calculate the ASA (<ASA>_calc_) corresponding to the average number of partners for the whole residue and the side chain. These estimates of average ASA compare very well with the means of the observed values (<ASA>_obs_) (Table 2); the correlation coefficient between the observed and calculated values is 0.99 for both the sets involving the whole residue and the side chain. It may be mentioned that other studies also report quite similar values for the mean ASA (absolute value, or that relative to the standard state) of residues [24,25]. Instead of the mean, some studies have used median ASA [24]. As such, median partner numbers (PN_M_) are also provided in Table 2.

**Table 2:** Partner number (median, PN_M _and average, <PN>), observed (<ASA>_obs_) and calculated (<ASA>_calc_) (corresponding to <PN>) ASAs for different amino acid residues

Residue	Whole Residue				Side Chain			
	PN_M_	<PN>	<ASA>_obs_	<ASA>_calc_	PN_M_	<PN>	<ASA>_obs_	<ASA>_calc_

Gly	7.5	7.4 (2.2)	26.6 (24.5)	25.9				

Ala	8.0	8.6 (2.5)	28.1 (30.9)	23.9	4.0	3.6 (1.9)	18.2 (21.2)	13.9

Ser	8.0	7.9 (2.6)	39.2 (33.2)	34.7	5.0	3.6 (2.0)	27.9 (24.2)	23.6

Cys	9.5	10.0 (2.3)	17.1 (21.0)	15.1	5.0	5.6 (1.7)	10.3 (15.7)	8.0

Thr	8.5	8.5 (2.6)	44.2 (36.0)	39.9	6.0	4.5 (2.2)	35.5 (30.0)	31.1

Asp	9.5	7.9 (2.5)	58.1 (37.2)	54.3	5.5	3.9 (2.1)	48.0 (30.6)	43.9

Pro	8.5	7.7 (2.6)	54.2 (39.5)	51.4	6.0	4.9 (2.4)	43.4 (32.8)	40.5

Asn	9.0	8.3 (2.7)	57.9 (40.8)	53.7	6.5	4.4 (2.3)	48.0 (33.7)	42.9

Val	9.5	10.3 (2.6)	24.1 (32.0)	20.3	5.5	6.0 (2.1)	19.0 (27.2)	13.5

Glu	9.5	8.4 (2.5)	73.4 (41.9)	70.7	5.5	4.0 (2.2)	63.8 (36.3)	59.9

Gln	9.0	9.0 (2.7)	68.6 (43.3)	62.1	6.5	4.6 (2.4)	59.6 (37.7)	54.6

His	9.5	9.7 (2.9)	53.8 (44.6)	48.2	6.0	5.7 (2.5)	46.5 (38.7)	40.1

Leu	9.5	11.0 (2.7)	28.8 (38.0)	21.9	6.5	6.6 (2.3)	23.3 (32.6)	16.9

Ile	8.5	11.0 (2.7)	25.0 (35.2)	18.9	6.5	6.9 (2.2)	20.5 (30.6)	15.2

Met	9.5	11.2 (3.1)	35.5 (45.8)	26.5	6.5	6.7 (2.6)	28.9 (38.1)	21.1

Lys	9.5	8.4 (2.4)	95.8 (42.9)	92.5	6.5	4.0 (2.1)	85.4 (37.0)	82.3

Phe	10.0	11.9 (2.9)	31.0 (39.8)	24.5	7.0	7.9 (2.5)	25.3 (35.1)	18.5

Tyr	10.0	11.5 (3.1)	45.5 (45.0)	38.2	7.5	7.5 (2.6)	39.7 (40.2)	33.8

Arg	11.0	10.1 (3.1)	85.5 (53.3)	80.0	8.0	5.8 (2.7)	77.3 (48.0)	70.4

Trp	11.0	12.6 (3.2)	43.5 (47.6)	36.9	8.0	8.8 (2.9)	37.8 (43.7)	29.3

The standard deviations are in parentheses. <ASA>_calc _at a given <PN> is obtained using the equation provided in Table 1.

We also compared the observed ASA (ASA_obs_) of a structure to ASA_calc_, calculated by considering each residue in the structure in turn and calculating its PN, which was then employed to derive its ASA using the equation given in Table 1. Two metrics were computed: *R*_A _= Σ ASA_i(calc)_/Σ ASA_i(obs) _(summed over all the residues, *i*), and



**Figure 4:**
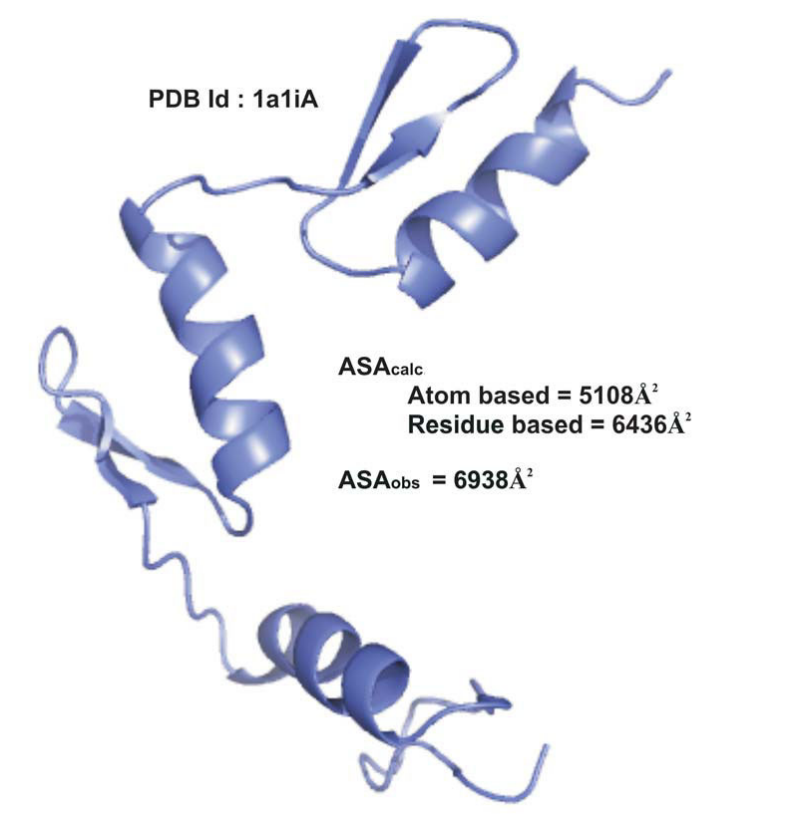
**Cartoon representation of a molecule showing the match between the observed and calculated ASA**.

These values were also calculated when the partner number was atom-based (i.e., the number of surrounding atoms) and an exponential equation was used to relate PN to ASA_calc _[7]. Results presented in Table 3 indicate that *R*_A _is closer to 1 and *R*_D _is smaller when the partner number is based on residues, suggesting a closer match with ASA_obs _for the residue-based, rather than the atom-based calculation. This can also be seen from the smaller difference, (ASA_calc _– ASA_obs_), for the former (Additional file 1, Figure S2). For the majority of the structures ASA_obs _is greater than ASA_calc _(individual values are available in Table S1, Additional file 1). We have identified at least one feature that may be responsible for the greater discrepancy involving the atom-based calculation – structures that are less compact, being made up of smaller domains, Figure 4 showing an example, the residue-based ASA_calc _is closer to ASA_obs_.

**Table 3:** Parameters indicating the match between observed and calculated ASA values in protein structures

	Calculations based on the number of	
Parameters	Partner atoms	Partner residues

*R*_A_	0.93 (0.11)	0.98 (0.07)

*R*_D_	0.37 (0.07)	0.30 (0.06)

Average values based on 275 structures (considering only the well-ordered structures, as given in Methods, and excluding those with missing atoms/residues).

### The webserver, ContPlot

**Figure 5:**
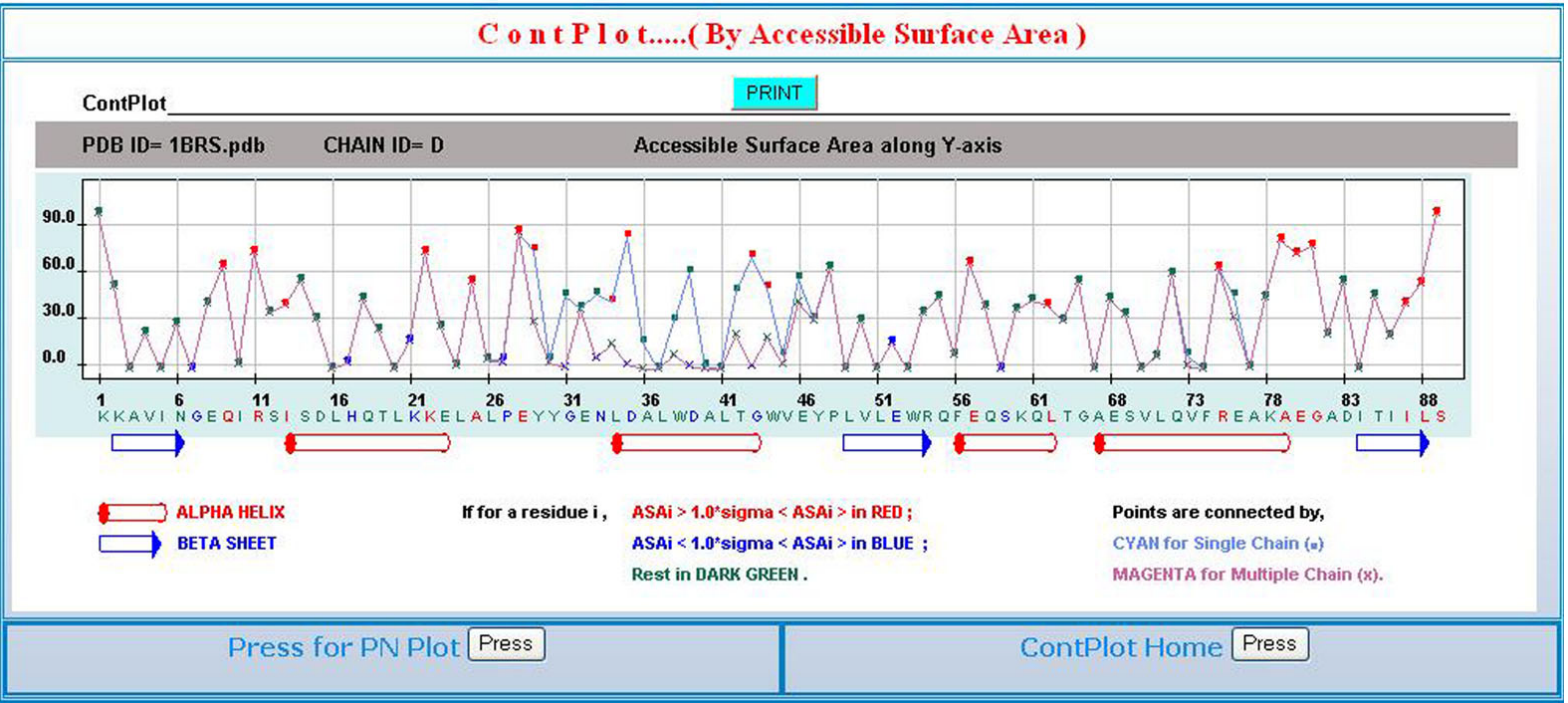
**A display from ContPlot for the 89 residue long inhibitor (barstar) in complexation with the enzyme barnase (PDB file**, 1brs) [34]** showing the relative accessibilities of the residues**. The values that are above (or below) the average value (by one standard deviation) for the same residue type have their symbols and the residue codes colored red (or blue); others (within ± 1σ) are in green. When two plots are overlaid (as in here) with the calculations done once for the chain in the isolated state and then in presence of another subunit in the complex, the coloring of the residue labels corresponds to the results from the latter calculations. Helices and β-strands are shown as red cylinders and blue arrows, respectively.

**Figure 6:**
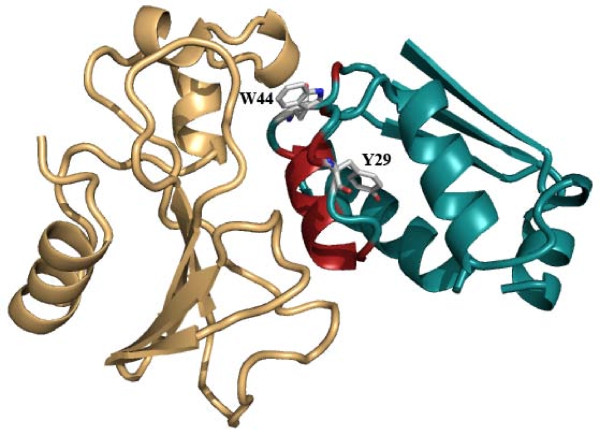
**A molecular representation of barnase-barstar complex (chain ID A in gold and D in cyan in the PDB file**, 1brs) [34]** with the interface region of barstar shown in red and the side chains of two hydrophobic residues Tyr29 and Trp44 indicated in stick**.

Having computed the average values of the ASA and PN for twenty residue types we felt that a software that would display the two parameters relative to the average values for all the residues in a polypeptide chain would be useful for the analysis of residue packing in relation to the secondary structures and the overall fold of the protein molecule. ContPlot is such a webserver that creates a plot based on either of the two parameters. For ASA, though the observed value is checked against average ASA value of a particular residue, what is plotted is the relative accessibility (i.e., what percentage of the surface area of residue (X) is accessible to solvent relative to its value in the standard state, taken as the ASA of X in the tripeptide, Ala-X-Ala (in the program NACCESS), so that all the residues are displayed in the range 0–100. The plot enables one to visually identify residues that are tightly packed, but the presence of hydrophobic residues that are less than optimally packed on the surface may indicate a binding site for another protein molecule. Indeed there is an option in the server to simultaneously display the results from another calculation including other chain(s) in a binary (or higher oligomeric) complex, thus providing a method to visually identify the residues in the interface [26]. Figure 5 shows the ContPlot (using ASA) of barstar alone and in complexation with barnase. Residues 27–46 constitute the longest segment of the polypeptide chain that forms the interface [27]. For this stretch there is a substantial decrease in ASA of residues on complexation. In the isolated state residue Tyr29 (red square) has an ASA value that is greater than 1.0σ times the average ASA of Tyr. There are quite a few residues of this type in the above stretch of the chain (Figure 6). When the calculation is done on the basis of PN, many of these residues would also be shown in red square, indicating a value of the PN that is smaller than 1.0σ times the average PN of the same residue type. In the complex state many of the residues in this stretch are shown in green (or blue) crosses, indicating the ASA values to be within (or less than) 1.0σ of the average value. Although 1.0σ level has been used here, the σ values are rather large (Table 2) and it may be easier to identify the deviant residues at a lower level. Many other issues are discussed in the help file associated with the server.

## Conclusion

A parameter, PN has been defined to indicate how many residues are found with 4.5 Å of a given residue. The histogram of the variation of PN has a skewness that indicates the hydrophobic or hydrophilic nature of the residue (Figures 1 and 2). ASA of a residue decreases with the increase in PN and a cubic equation has been used to represent the dependence (Table 1, Figures 3 and S1, Additional file 1). The average values of ASA and PN associated with the residues have been used to develop a webserver, ContPlot that reads a PDB file to display the deviation of these values from the average values of the corresponding residues along the protein sequence (Figure 5). Extending the concept that each residue in a protein sequence would try to achieve a value of ASA or PN that is close to its average value we have devised an algorithm that can identify the native fold from a set of decoys representing near-native structures (Bahadur and Chakrabarti, unpublished). Furthermore, the presence of cluster of hydrophobic residues with unsatisfied (i.e., below-average) PNs (or above-average ASAs) (Figure 5) could indicate the existence of a protein-protein interaction site and this is being used to identify interface patches in protein structures (Pal and Chakrabarti, unpublished).

## Methods

Atomic coordinates were obtained from the Protein Data Bank (PDB) [28]. The analysis was carried out using the same dataset of 432 polypeptide chains used in [7] and the same criteria on B-factor were used to select only the well-ordered residues. The solvent accessible surface area (ASA) was computed using the program NACCESS [29], which is an implementation of the algorithm by Lee and Richards [30], using the default probe radius of 1.4 Å. This ASA has been designated as the observed value (ASA_obs_) to distinguish it from the one calculated (ASA_calc_) using the equation derived in this paper. Calculations were restricted to the atoms of a particular subunit only. Two residues are in contact if any atom-to-atom distance between the pair are within 4.5 Å [31]. The partner number of a residue is the number of other residues within a distance of 4.5 Å from any atom of the residue under consideration; the flanking residues were not considered as partner if the interaction was only with the main-chain atoms. The secondary structures were determined using DSSP [32]. The webserver, ContPlot uses the average values of ASA and PN (and the associated standard deviations) for all the residues given in Table 2 and is available, along with necessary documentation at: .

## Authors' contributions

PC conceptualized the work that was carried out by AP, RPB and PSR. AP, RPB and PC participated in interpretation of the data and writing the manuscript. All the authors have read and accepted the final version of the manuscript.

## Supplementary Material

Additional File 1 **Supplementary data**. Table S1. Observed and calculated ASAs, and the match between them, in different protein structures. Figure S1. Variation of the mean accessible surface area (Å^2^) with partner number for residues other than Lys and Met. Figure S2. Plot of the difference, (ASA_calc _– ASA_obs_), for 275 individual PDB files. Click here for file
