# Supplementary material for: Accessibility and partner number of protein residues, their relationship and a webserver, ContPlot for their display
Source: BMC Bioinformatics. 2009 Apr 8;10:103. doi: 10.1186/1471-2105-10-103 (PMC2680847; doi:10.1186/1471-2105-10-103)

Table 4. Observed and calculated ASAs, and the match between them, in different protein structures

| **PDB ID** | **R_A_ (Atom-based)** | **R_A_**  **(Res-based)** | **R_D_ (Atom-based)** | **R_D_**  **(Res-based)** | **ASA_calc._**  **(Atom-based) in Å^2^** | **ASA_calc._**  **(Res-based) in Å^2^** | **ASA_obs_. in Å^2^** |
| --- | --- | --- | --- | --- | --- | --- | --- |
| 153l_ | 0.95 | 0.97 | 0.38 | 0.32 | 7890 | 8031 | 8264 |
| 16pk_ | 1.00 | 1.00 | 0.44 | 0.34 | 17918 | 17809 | 17887 |
| 19hcA | 0.86 | 0.95 | 0.28 | 0.24 | 16129 | 17867 | 18837 |
| 1a1iA | 0.74 | 0.93 | 0.30 | 0.19 | 5108 | 6436 | 6939 |
| 1a1yI | 0.85 | 0.96 | 0.34 | 0.27 | 3652 | 4142 | 4314 |
| 1a2pA | 0.92 | 1.01 | 0.35 | 0.30 | 5503 | 6022 | 5978 |
| 1a2zA | 0.99 | 1.00 | 0.38 | 0.33 | 10139 | 10227 | 10236 |
| 1a34A | 0.90 | 0.97 | 0.29 | 0.24 | 8743 | 9379 | 9670 |
| 1a4iB | 0.94 | 0.97 | 0.41 | 0.33 | 13317 | 13753 | 14127 |
| 1a6m_ | 0.84 | 0.92 | 0.37 | 0.29 | 6651 | 7338 | 7942 |
| 1aba_ | 0.84 | 1.00 | 0.35 | 0.27 | 4588 | 5429 | 5449 |
| 1adoA | 1.02 | 1.03 | 0.43 | 0.34 | 16803 | 16918 | 16501 |
| 1alvA | 0.81 | 0.90 | 0.37 | 0.27 | 8544 | 9520 | 10524 |
| 1amm_ | 0.94 | 1.06 | 0.35 | 0.31 | 8113 | 9133 | 8608 |
| 1amx_ | 0.98 | 1.05 | 0.35 | 0.26 | 7383 | 7877 | 7505 |
| 1apyA | 0.82 | 0.93 | 0.32 | 0.26 | 8331 | 9370 | 10112 |
| 1aqb_ | 0.96 | 1.01 | 0.35 | 0.30 | 9336 | 9774 | 9711 |
| 1arv_ | 1.06 | 1.04 | 0.45 | 0.35 | 14260 | 14012 | 13415 |
| 1atlA | 0.97 | 0.99 | 0.43 | 0.32 | 9076 | 9332 | 9387 |
| 1aun_ | 1.00 | 1.03 | 0.37 | 0.31 | 9477 | 9754 | 9493 |
| 1axn_ | 0.95 | 1.01 | 0.42 | 0.32 | 14357 | 15233 | 15095 |
| 1ay7B | 0.90 | 1.02 | 0.37 | 0.31 | 4385 | 4951 | 4857 |
| 1ayfA | 0.93 | 0.97 | 0.34 | 0.25 | 5283 | 5544 | 5689 |
| 1b0nB | 0.65 | 0.83 | 0.35 | 0.17 | 2131 | 2720 | 3279 |
| 1b0uA | 0.99 | 1.04 | 0.42 | 0.33 | 11991 | 12643 | 12118 |
| 1b0yA | 0.86 | 0.96 | 0.32 | 0.27 | 4261 | 4733 | 4938 |
| 1b16A | 0.97 | 1.03 | 0.41 | 0.31 | 11660 | 12422 | 12033 |
| 1b2vA | 0.91 | 0.90 | 0.40 | 0.33 | 7503 | 7381 | 8232 |
| 1b3aA | 0.85 | 0.97 | 0.30 | 0.24 | 4194 | 4786 | 4927 |
| 1b5eA | 0.94 | 0.99 | 0.38 | 0.31 | 11604 | 12170 | 12316 |
| 1b7cA | 0.86 | 0.93 | 0.28 | 0.24 | 3570 | 3844 | 4139 |
| 1b8oA | 1.04 | 1.11 | 0.45 | 0.37 | 12502 | 13288 | 11977 |
| 1babB | 0.82 | 0.88 | 0.38 | 0.28 | 6435 | 6890 | 7870 |
| 1bbhA | 0.80 | 0.91 | 0.34 | 0.28 | 5966 | 6803 | 7486 |
| 1bdo_ | 0.93 | 0.94 | 0.30 | 0.22 | 4553 | 4612 | 4921 |
| 1benB | 0.76 | 0.86 | 0.25 | 0.25 | 2367 | 2692 | 3113 |
| 1bfg_ | 1.03 | 1.05 | 0.35 | 0.29 | 6621 | 6785 | 6455 |
| 1bgf_ | 0.78 | 0.87 | 0.39 | 0.28 | 6148 | 6842 | 7877 |
| 1bj7_ | 0.99 | 1.04 | 0.38 | 0.29 | 7262 | 7607 | 7315 |
| 1bk7A | 0.97 | 0.99 | 0.35 | 0.28 | 8898 | 9014 | 9141 |
| 1bkrA | 0.85 | 0.93 | 0.37 | 0.26 | 5149 | 5660 | 6062 |
| 1brt_ | 1.04 | 1.03 | 0.49 | 0.37 | 11431 | 11309 | 11033 |
| 1bs4A | 0.94 | 0.97 | 0.34 | 0.29 | 8769 | 9073 | 9372 |
| 1bs9_ | 0.99 | 0.93 | 0.47 | 0.41 | 8023 | 7541 | 8133 |
| 1bsmA | 0.90 | 0.97 | 0.39 | 0.31 | 9079 | 9790 | 10122 |
| 1btn_ | 0.92 | 1.01 | 0.31 | 0.24 | 5877 | 6429 | 6367 |
| 1bu7A | 1.04 | 1.06 | 0.44 | 0.35 | 20315 | 20669 | 19559 |
| 1bx4A | 1.03 | 1.05 | 0.41 | 0.33 | 16227 | 16486 | 15685 |
| 1bxaA | 0.98 | 0.97 | 0.32 | 0.28 | 5361 | 5291 | 5443 |
| 1bxoA | 1.08 | 1.03 | 0.43 | 0.36 | 13836 | 13246 | 12862 |
| 1byi_ | 0.97 | 0.99 | 0.42 | 0.31 | 10422 | 10621 | 10779 |
| 1byqA | 0.98 | 1.02 | 0.37 | 0.28 | 10532 | 10880 | 10715 |
| 1byrA | 0.95 | 0.97 | 0.40 | 0.33 | 6716 | 6846 | 7058 |
| 1c3d_ | 1.03 | 0.99 | 0.43 | 0.34 | 12490 | 12085 | 12164 |
| 1c52_ | 0.92 | 0.97 | 0.32 | 0.30 | 6579 | 6973 | 7186 |
| 1cc8A | 0.89 | 0.97 | 0.32 | 0.21 | 3866 | 4232 | 4359 |
| 1cewI | 0.89 | 0.95 | 0.28 | 0.23 | 6254 | 6610 | 6992 |
| 1cex_ | 0.98 | 0.96 | 0.44 | 0.35 | 8205 | 8000 | 8360 |
| 1cf9A | 1.06 | 1.11 | 0.43 | 0.37 | 35782 | 37327 | 33728 |
| 1cfb_ | 0.93 | 0.95 | 0.33 | 0.28 | 10200 | 10401 | 10926 |
| 1ckaA | 0.87 | 0.99 | 0.27 | 0.23 | 3434 | 3916 | 3954 |
| 1cleA | 1.14 | 1.10 | 0.54 | 0.45 | 21429 | 20556 | 18765 |
| 1cmbA | 0.83 | 0.96 | 0.32 | 0.27 | 6199 | 7134 | 7443 |
| 1cozA | 0.90 | 0.99 | 0.35 | 0.27 | 6557 | 7250 | 7326 |
| 1cpo_ | 1.02 | 1.04 | 0.42 | 0.37 | 13402 | 13699 | 13176 |
| 1cpq_ | 0.77 | 0.83 | 0.37 | 0.29 | 5636 | 6066 | 7289 |
| 1cqyA | 0.92 | 0.99 | 0.33 | 0.26 | 5221 | 5622 | 5675 |
| 1ctj_ | 0.86 | 0.98 | 0.31 | 0.25 | 4479 | 5156 | 5236 |
| 1ctqA | 0.97 | 1.03 | 0.38 | 0.29 | 7986 | 8481 | 8230 |
| 1cv8_ | 0.98 | 0.97 | 0.41 | 0.31 | 8446 | 8386 | 8639 |
| 1cy5A | 0.84 | 0.91 | 0.38 | 0.26 | 4259 | 4626 | 5087 |
| 1cydA | 0.96 | 0.94 | 0.43 | 0.31 | 10543 | 10368 | 11012 |
| 1cyo_ | 0.83 | 0.93 | 0.33 | 0.26 | 4916 | 5532 | 5918 |
| 1czfA | 1.04 | 1.05 | 0.43 | 0.37 | 12760 | 12817 | 12252 |
| 1czpA | 0.94 | 0.98 | 0.36 | 0.30 | 4742 | 4976 | 5067 |
| 1d3vA | 1.09 | 1.08 | 0.48 | 0.38 | 13555 | 13398 | 12452 |
| 1d7pM | 0.97 | 1.03 | 0.37 | 0.32 | 8041 | 8584 | 8306 |
| 1dciA | 0.90 | 0.94 | 0.42 | 0.34 | 12565 | 13167 | 13982 |
| 1dg9A | 0.90 | 0.98 | 0.40 | 0.30 | 7434 | 8048 | 8215 |
| 1dhn_ | 0.91 | 0.99 | 0.32 | 0.25 | 6681 | 7316 | 7366 |
| 1din_ | 1.00 | 1.04 | 0.48 | 0.36 | 9932 | 10360 | 9940 |
| 1dokA | 0.85 | 0.92 | 0.30 | 0.21 | 4707 | 5135 | 5556 |
| 1dosA | 0.96 | 1.00 | 0.40 | 0.32 | 16515 | 17129 | 17144 |
| 1dozA | 0.97 | 1.00 | 0.42 | 0.34 | 13706 | 14069 | 14118 |
| 1dpsD | 0.86 | 0.92 | 0.40 | 0.28 | 7270 | 7810 | 8482 |
| 1dptA | 0.86 | 0.94 | 0.37 | 0.28 | 5796 | 6298 | 6709 |
| 1dun_ | 0.95 | 1.04 | 0.30 | 0.20 | 7705 | 8428 | 8089 |
| 1dxgA | 0.85 | 0.88 | 0.19 | 0.18 | 2247 | 2330 | 2643 |
| 1ecpA | 0.99 | 1.04 | 0.44 | 0.32 | 10939 | 11424 | 11006 |
| 1edg_ | 1.08 | 1.06 | 0.49 | 0.39 | 16659 | 16384 | 15430 |
| 1edmB | 0.76 | 0.85 | 0.26 | 0.21 | 2297 | 2549 | 3013 |
| 1egpA | 0.75 | 0.94 | 0.27 | 0.20 | 2897 | 3665 | 3879 |
| 1eus_ | 1.16 | 1.11 | 0.51 | 0.38 | 15594 | 14873 | 13407 |
| 1extB | 0.83 | 0.91 | 0.26 | 0.23 | 9413 | 10343 | 11318 |
| 1fce_ | 1.18 | 1.13 | 0.50 | 0.43 | 25422 | 24486 | 21634 |
| 1fipA | 0.74 | 0.87 | 0.32 | 0.22 | 4235 | 4980 | 5752 |
| 1fltV | 0.84 | 0.94 | 0.24 | 0.19 | 5856 | 6581 | 6972 |
| 1fna_ | 0.93 | 0.96 | 0.28 | 0.23 | 5021 | 5183 | 5397 |
| 1gdoB | 1.07 | 1.11 | 0.45 | 0.36 | 10758 | 11216 | 10071 |
| 1gof_ | 1.20 | 1.13 | 0.54 | 0.43 | 25925 | 24434 | 21586 |
| 1gpeA | 1.12 | 1.07 | 0.54 | 0.45 | 21876 | 20983 | 19609 |
| 1gsa_ | 1.07 | 1.10 | 0.45 | 0.36 | 14665 | 15048 | 13646 |
| 1guqA | 0.98 | 1.06 | 0.40 | 0.35 | 16304 | 17566 | 16559 |
| 1hfc_ | 0.96 | 1.07 | 0.36 | 0.27 | 7617 | 8503 | 7968 |
| 1hfeS | 0.69 | 0.88 | 0.32 | 0.21 | 6029 | 7692 | 8777 |
| 1hka_ | 0.94 | 0.98 | 0.36 | 0.28 | 8091 | 8384 | 8582 |
| 1hleB | 0.77 | 0.94 | 0.23 | 0.15 | 2585 | 3187 | 3375 |
| 1hoe_ | 0.83 | 0.92 | 0.35 | 0.25 | 3554 | 3958 | 4305 |
| 1iab_ | 0.97 | 1.04 | 0.39 | 0.33 | 8902 | 9521 | 9168 |
| 1idaA | 0.95 | 1.00 | 0.29 | 0.20 | 6302 | 6652 | 6654 |
| 1ifc_ | 0.98 | 1.01 | 0.32 | 0.29 | 6891 | 7086 | 7034 |
| 1iibA | 0.85 | 0.90 | 0.38 | 0.29 | 5058 | 5361 | 5930 |
| 1isuA | 0.81 | 0.92 | 0.27 | 0.26 | 3178 | 3576 | 3900 |
| 1ixh_ | 1.03 | 1.06 | 0.44 | 0.34 | 13736 | 14032 | 13282 |
| 1jdw_ | 1.13 | 1.15 | 0.55 | 0.43 | 15233 | 15493 | 13471 |
| 1knb_ | 0.99 | 0.98 | 0.38 | 0.29 | 8839 | 8717 | 8890 |
| 1koe_ | 1.01 | 1.02 | 0.43 | 0.33 | 7943 | 8000 | 7880 |
| 1kp6A | 0.78 | 0.82 | 0.34 | 0.26 | 3708 | 3935 | 4771 |
| 1kptA | 0.91 | 0.95 | 0.36 | 0.27 | 4684 | 4881 | 5156 |
| 1kveA | 0.81 | 0.93 | 0.25 | 0.18 | 4328 | 4972 | 5340 |
| 1kveB | 0.81 | 0.88 | 0.25 | 0.22 | 4321 | 4723 | 5350 |
| 1lam_ | 1.09 | 1.09 | 0.46 | 0.37 | 21412 | 21402 | 19687 |
| 1lcl_ | 1.06 | 1.05 | 0.37 | 0.30 | 7443 | 7392 | 7014 |
| 1lkkA | 0.93 | 1.03 | 0.32 | 0.27 | 5956 | 6643 | 6428 |
| 1louA | 0.94 | 0.99 | 0.33 | 0.24 | 5767 | 6067 | 6120 |
| 1mai_ | 0.93 | 1.03 | 0.32 | 0.27 | 6229 | 6935 | 6730 |
| 1mdc_ | 0.98 | 0.98 | 0.29 | 0.27 | 6838 | 6824 | 6969 |
| 1mgtA | 0.99 | 1.03 | 0.37 | 0.32 | 8094 | 8400 | 8142 |
| 1mkaA | 0.98 | 1.03 | 0.35 | 0.25 | 8555 | 8920 | 8686 |
| 1mla_ | 1.04 | 0.98 | 0.47 | 0.34 | 12502 | 11720 | 11978 |
| 1mml_ | 0.97 | 1.02 | 0.40 | 0.30 | 12417 | 13024 | 12778 |
| 1mof_ | 0.64 | 0.80 | 0.37 | 0.25 | 3162 | 3935 | 4910 |
| 1molA | 0.90 | 0.99 | 0.30 | 0.23 | 5343 | 5822 | 5904 |
| 1moq_ | 1.05 | 1.04 | 0.49 | 0.37 | 15512 | 15360 | 14711 |
| 1mrj_ | 0.98 | 0.99 | 0.41 | 0.33 | 10925 | 11025 | 11163 |
| 1mroB | 1.05 | 0.99 | 0.44 | 0.36 | 19316 | 18158 | 18400 |
| 1mroC | 0.93 | 1.02 | 0.37 | 0.29 | 12969 | 14211 | 13932 |
| 1msi_ | 0.90 | 0.88 | 0.34 | 0.25 | 3281 | 3185 | 3638 |
| 1msk_ | 0.99 | 1.04 | 0.44 | 0.35 | 15143 | 15919 | 15269 |
| 1mtyB | 0.94 | 0.99 | 0.41 | 0.33 | 17354 | 18267 | 18403 |
| 1mtyG | 0.81 | 0.91 | 0.35 | 0.26 | 8350 | 9454 | 10336 |
| 1mugA | 0.97 | 1.02 | 0.40 | 0.32 | 8257 | 8732 | 8531 |
| 1mun_ | 0.90 | 0.94 | 0.39 | 0.31 | 9902 | 10313 | 10956 |
| 1nar_ | 1.04 | 1.05 | 0.47 | 0.36 | 13656 | 13695 | 13073 |
| 1nbcA | 1.02 | 1.03 | 0.34 | 0.28 | 7253 | 7295 | 7109 |
| 1ncoA | 0.91 | 0.92 | 0.33 | 0.28 | 5445 | 5463 | 5951 |
| 1nif_ | 1.06 | 1.05 | 0.43 | 0.34 | 16384 | 16281 | 15442 |
| 1nkd_ | 0.63 | 0.77 | 0.41 | 0.30 | 2837 | 3441 | 4496 |
| 1nkr_ | 0.95 | 0.97 | 0.30 | 0.28 | 9838 | 10052 | 10360 |
| 1nls_ | 1.08 | 1.09 | 0.40 | 0.35 | 11477 | 11544 | 10621 |
| 1nox_ | 0.92 | 0.95 | 0.38 | 0.28 | 10988 | 11317 | 11962 |
| 1np4A | 0.99 | 1.00 | 0.36 | 0.26 | 9722 | 9815 | 9780 |
| 1npk_ | 0.88 | 0.94 | 0.38 | 0.29 | 7058 | 7555 | 8038 |
| 1oaa_ | 0.96 | 0.96 | 0.44 | 0.33 | 11767 | 11728 | 12277 |
| 1opd_ | 0.91 | 0.90 | 0.35 | 0.28 | 4382 | 4349 | 4813 |
| 1otfA | 0.77 | 0.90 | 0.30 | 0.23 | 3528 | 4131 | 4607 |
| 1pbe_ | 1.06 | 1.07 | 0.46 | 0.37 | 18044 | 18227 | 16981 |
| 1pcfA | 0.73 | 0.91 | 0.32 | 0.23 | 4003 | 4979 | 5493 |
| 1pdo_ | 0.91 | 0.94 | 0.36 | 0.28 | 6345 | 6566 | 6994 |
| 1plc_ | 0.94 | 0.97 | 0.30 | 0.24 | 4759 | 4884 | 5051 |
| 1pne_ | 0.93 | 0.93 | 0.39 | 0.29 | 6443 | 6473 | 6965 |
| 1poa_ | 0.83 | 0.91 | 0.32 | 0.26 | 5632 | 6185 | 6797 |
| 1poc_ | 0.83 | 0.96 | 0.32 | 0.26 | 6450 | 7398 | 7741 |
| 1ppn_ | 1.01 | 1.07 | 0.44 | 0.35 | 9540 | 10076 | 9421 |
| 1psrA | 0.77 | 0.86 | 0.36 | 0.28 | 5143 | 5781 | 6711 |
| 1ptq_ | 0.82 | 0.88 | 0.28 | 0.19 | 2939 | 3160 | 3587 |
| 1pty_ | 1.03 | 1.06 | 0.44 | 0.36 | 13668 | 14004 | 13229 |
| 1qb7A | 0.90 | 0.95 | 0.35 | 0.26 | 11982 | 12570 | 13291 |
| 1qcxA | 1.12 | 1.07 | 0.46 | 0.40 | 14878 | 14152 | 13251 |
| 1qgiA | 0.99 | 1.05 | 0.41 | 0.37 | 11610 | 12229 | 11685 |
| 1qh4A | 0.99 | 1.02 | 0.44 | 0.35 | 17145 | 17674 | 17380 |
| 1qh5A | 1.04 | 0.99 | 0.45 | 0.35 | 11979 | 11488 | 11572 |
| 1qhfA | 1.01 | 1.05 | 0.42 | 0.32 | 11465 | 11863 | 11319 |
| 1qksA | 1.18 | 1.14 | 0.51 | 0.39 | 24284 | 23339 | 20526 |
| 1qq4A | 1.06 | 1.02 | 0.41 | 0.34 | 8345 | 8028 | 7840 |
| 1qq5A | 0.97 | 0.99 | 0.46 | 0.34 | 10765 | 10958 | 11045 |
| 1qsgA | 1.00 | 0.98 | 0.44 | 0.34 | 12228 | 12070 | 12276 |
| 1qtsA | 0.97 | 1.02 | 0.38 | 0.28 | 12444 | 13082 | 12776 |
| 1qtwA | 1.00 | 1.04 | 0.47 | 0.37 | 11952 | 12485 | 11964 |
| 1rb9_ | 0.84 | 0.92 | 0.29 | 0.27 | 2647 | 2911 | 3163 |
| 1rcf_ | 0.99 | 1.01 | 0.43 | 0.31 | 7683 | 7834 | 7724 |
| 1regY | 0.92 | 1.02 | 0.33 | 0.25 | 6628 | 7352 | 7242 |
| 1rgeA | 0.90 | 0.99 | 0.32 | 0.29 | 4980 | 5488 | 5518 |
| 1rie_ | 0.97 | 0.99 | 0.34 | 0.27 | 6374 | 6567 | 6601 |
| 1rzl_ | 0.85 | 0.88 | 0.38 | 0.30 | 4013 | 4161 | 4721 |
| 1sfp_ | 1.00 | 1.02 | 0.37 | 0.27 | 5960 | 6096 | 5962 |
| 1sgpI | 0.80 | 0.86 | 0.28 | 0.26 | 2744 | 2954 | 3419 |
| 1smd_ | 1.14 | 1.16 | 0.53 | 0.45 | 20121 | 20553 | 17698 |
| 1smlA | 0.98 | 0.97 | 0.43 | 0.32 | 11983 | 11780 | 12196 |
| 1sra_ | 0.86 | 0.95 | 0.38 | 0.26 | 7535 | 8368 | 8789 |
| 1sur_ | 0.90 | 0.97 | 0.41 | 0.31 | 10494 | 11326 | 11644 |
| 1svfA | 0.51 | 0.65 | 0.49 | 0.35 | 2805 | 3585 | 5552 |
| 1svfB | 0.60 | 0.75 | 0.40 | 0.26 | 2437 | 3046 | 4074 |
| 1svpA | 1.00 | 1.04 | 0.34 | 0.29 | 8529 | 8890 | 8568 |
| 1tafA | 0.65 | 0.79 | 0.37 | 0.24 | 3828 | 4675 | 5917 |
| 1taxA | 1.07 | 1.01 | 0.54 | 0.44 | 11930 | 11233 | 11162 |
| 1tgxA | 0.89 | 0.95 | 0.25 | 0.20 | 3879 | 4159 | 4359 |
| 1tib_ | 1.04 | 1.06 | 0.43 | 0.36 | 11648 | 11935 | 11239 |
| 1tif_ | 0.86 | 0.99 | 0.29 | 0.19 | 4602 | 5301 | 5334 |
| 1tl2A | 1.02 | 1.12 | 0.36 | 0.30 | 11032 | 12020 | 10775 |
| 1tml_ | 1.02 | 1.01 | 0.48 | 0.40 | 11749 | 11670 | 11563 |
| 1toaA | 0.97 | 0.96 | 0.45 | 0.34 | 11879 | 11802 | 12275 |
| 1ttbA | 0.98 | 1.00 | 0.33 | 0.29 | 6631 | 6780 | 6801 |
| 1tvxB | 0.86 | 0.94 | 0.29 | 0.24 | 4582 | 5027 | 5355 |
| 1u9aA | 0.88 | 0.95 | 0.38 | 0.31 | 7670 | 8322 | 8752 |
| 1ubpA | 0.80 | 0.87 | 0.36 | 0.28 | 5203 | 5661 | 6477 |
| 1ubpB | 0.90 | 0.98 | 0.28 | 0.22 | 7189 | 7811 | 7954 |
| 1unkA | 0.82 | 0.90 | 0.33 | 0.30 | 4560 | 5005 | 5545 |
| 1uox_ | 0.94 | 0.99 | 0.33 | 0.25 | 16094 | 17009 | 17111 |
| 1vcaA | 1.00 | 0.99 | 0.31 | 0.26 | 11190 | 11056 | 11181 |
| 1vfrA | 0.89 | 0.96 | 0.39 | 0.27 | 11042 | 11972 | 12462 |
| 1vfyA | 0.78 | 0.88 | 0.31 | 0.25 | 3623 | 4082 | 4621 |
| 1vhh_ | 0.94 | 1.03 | 0.35 | 0.30 | 7260 | 7970 | 7759 |
| 1vid_ | 1.02 | 1.00 | 0.44 | 0.36 | 9622 | 9502 | 9474 |
| 1vie_ | 0.85 | 0.96 | 0.28 | 0.23 | 3340 | 3801 | 3948 |
| 1vsrA | 0.89 | 0.95 | 0.38 | 0.30 | 6980 | 7475 | 7884 |
| 1wab_ | 0.95 | 1.01 | 0.43 | 0.34 | 9204 | 9749 | 9696 |
| 1wapB | 0.89 | 0.93 | 0.24 | 0.19 | 4118 | 4285 | 4601 |
| 1whi_ | 0.99 | 1.03 | 0.33 | 0.26 | 6902 | 7161 | 6942 |
| 1who_ | 0.95 | 0.97 | 0.32 | 0.25 | 5245 | 5330 | 5501 |
| 1xnb_ | 1.06 | 1.04 | 0.41 | 0.34 | 8270 | 8171 | 7823 |
| 1yacA | 0.89 | 0.95 | 0.45 | 0.33 | 9104 | 9726 | 10261 |
| 1yge_ | 1.11 | 1.10 | 0.48 | 0.37 | 36985 | 36834 | 33444 |
| 256bA | 0.79 | 0.87 | 0.36 | 0.29 | 5006 | 5498 | 6311 |
| 2abk_ | 0.94 | 1.00 | 0.37 | 0.28 | 10020 | 10716 | 10673 |
| 2acy_ | 0.92 | 0.99 | 0.33 | 0.29 | 5209 | 5635 | 5675 |
| 2ayh_ | 1.06 | 1.09 | 0.39 | 0.32 | 9870 | 10163 | 9321 |
| 2bopA | 0.83 | 0.93 | 0.31 | 0.23 | 4716 | 5244 | 5669 |
| 2bosA | 0.86 | 0.88 | 0.32 | 0.24 | 3518 | 3598 | 4091 |
| 2cbp_ | 0.91 | 0.97 | 0.33 | 0.24 | 4742 | 5053 | 5223 |
| 2chsA | 0.94 | 1.01 | 0.33 | 0.23 | 6485 | 6931 | 6869 |
| 2cpgA | 0.71 | 0.84 | 0.31 | 0.21 | 3115 | 3654 | 4365 |
| 2ctc_ | 1.05 | 1.06 | 0.47 | 0.39 | 12325 | 12456 | 11719 |
| 2dri_ | 1.02 | 1.02 | 0.44 | 0.34 | 11717 | 11763 | 11512 |
| 2ebn_ | 1.05 | 1.03 | 0.45 | 0.35 | 12181 | 11982 | 11644 |
| 2end_ | 0.86 | 0.97 | 0.38 | 0.27 | 6780 | 7697 | 7919 |
| 2erl_ | 0.69 | 0.80 | 0.34 | 0.25 | 1999 | 2321 | 2912 |
| 2fdn_ | 0.85 | 0.91 | 0.26 | 0.25 | 2772 | 2977 | 3267 |
| 2gdm_ | 0.83 | 0.88 | 0.37 | 0.28 | 6573 | 6977 | 7885 |
| 2hbg_ | 0.84 | 0.87 | 0.37 | 0.33 | 6096 | 6369 | 7294 |
| 2igd_ | 0.83 | 0.93 | 0.27 | 0.21 | 3487 | 3904 | 4213 |
| 2knt_ | 0.84 | 1.03 | 0.26 | 0.21 | 3269 | 4011 | 3892 |
| 2lisA | 0.82 | 0.93 | 0.35 | 0.27 | 7100 | 8046 | 8658 |
| 2myr_ | 1.04 | 1.08 | 0.50 | 0.43 | 18796 | 19454 | 18038 |
| 2nlrA | 1.05 | 1.03 | 0.43 | 0.34 | 9224 | 9057 | 8755 |
| 2pii_ | 0.91 | 1.00 | 0.28 | 0.22 | 7173 | 7905 | 7885 |
| 2pspA | 0.85 | 0.96 | 0.28 | 0.26 | 5406 | 6073 | 6342 |
| 2pth_ | 0.95 | 0.97 | 0.41 | 0.33 | 8854 | 9042 | 9288 |
| 2rn2_ | 0.95 | 1.02 | 0.33 | 0.28 | 8273 | 8913 | 8701 |
| 2sak_ | 0.93 | 1.04 | 0.32 | 0.25 | 7247 | 8151 | 7813 |
| 2sn3_ | 0.87 | 0.88 | 0.30 | 0.24 | 3618 | 3680 | 4167 |
| 2sns_ | 0.98 | 1.12 | 0.34 | 0.29 | 7761 | 8877 | 7951 |
| 2tpsA | 1.00 | 1.01 | 0.42 | 0.33 | 10539 | 10715 | 10564 |
| 2trxA | 0.98 | 0.98 | 0.36 | 0.28 | 5623 | 5641 | 5766 |
| 2tysB | 1.11 | 1.07 | 0.54 | 0.42 | 16180 | 15513 | 14541 |
| 3chbD | 0.88 | 0.92 | 0.32 | 0.26 | 5740 | 6007 | 6552 |
| 3chy_ | 0.96 | 1.01 | 0.40 | 0.32 | 6233 | 6560 | 6510 |
| 3cyr_ | 0.84 | 0.94 | 0.25 | 0.20 | 7218 | 8028 | 8543 |
| 3eng_ | 1.03 | 1.02 | 0.40 | 0.32 | 9403 | 9356 | 9139 |
| 3ezmA | 0.78 | 0.92 | 0.24 | 0.15 | 6803 | 7979 | 8677 |
| 3grs_ | 1.00 | 1.00 | 0.40 | 0.30 | 22275 | 22464 | 22386 |
| 3lzt_ | 0.90 | 0.92 | 0.34 | 0.29 | 5690 | 5833 | 6332 |
| 3pte_ | 1.09 | 1.01 | 0.49 | 0.41 | 14186 | 13109 | 13030 |
| 3pviA | 0.87 | 0.97 | 0.36 | 0.28 | 8559 | 9546 | 9799 |
| 3pyp_ | 0.99 | 1.04 | 0.39 | 0.27 | 6304 | 6628 | 6390 |
| 3sdhA | 0.83 | 0.86 | 0.38 | 0.30 | 6401 | 6574 | 7666 |
| 3sil_ | 1.16 | 1.10 | 0.60 | 0.54 | 16557 | 15683 | 14240 |
| 3tss_ | 0.97 | 1.02 | 0.35 | 0.27 | 9263 | 9799 | 9571 |
| 3vub_ | 0.89 | 0.96 | 0.30 | 0.21 | 5766 | 6247 | 6489 |
| 451c_ | 0.87 | 0.93 | 0.29 | 0.23 | 4173 | 4445 | 4773 |
| 4eugA | 0.98 | 1.02 | 0.39 | 0.32 | 10360 | 10812 | 10604 |
| 6cel_ | 1.09 | 1.02 | 0.46 | 0.39 | 17736 | 16612 | 16207 |
| 6gsvA | 0.94 | 1.03 | 0.41 | 0.32 | 10143 | 11202 | 10830 |
| 7a3hA | 1.05 | 1.04 | 0.53 | 0.41 | 11967 | 11918 | 11418 |
| 7rsa_ | 0.90 | 0.92 | 0.34 | 0.26 | 6259 | 6422 | 6984 |

Figure 7. Variation of the mean accessible surface area (Å^2^) with partner number for residues other than Lys and Met. The curves corresponding to the best least-squares fit, y = b_0_ + b_1_x + b_2_x^2^ + b_3_x^3^ (with b_0_, b_1_, b_2_ and b_3_ given in Table 1) are also shown. There are two plots for each residue (except Gly) – for the whole residue (w) and for the side chain (sc) only.


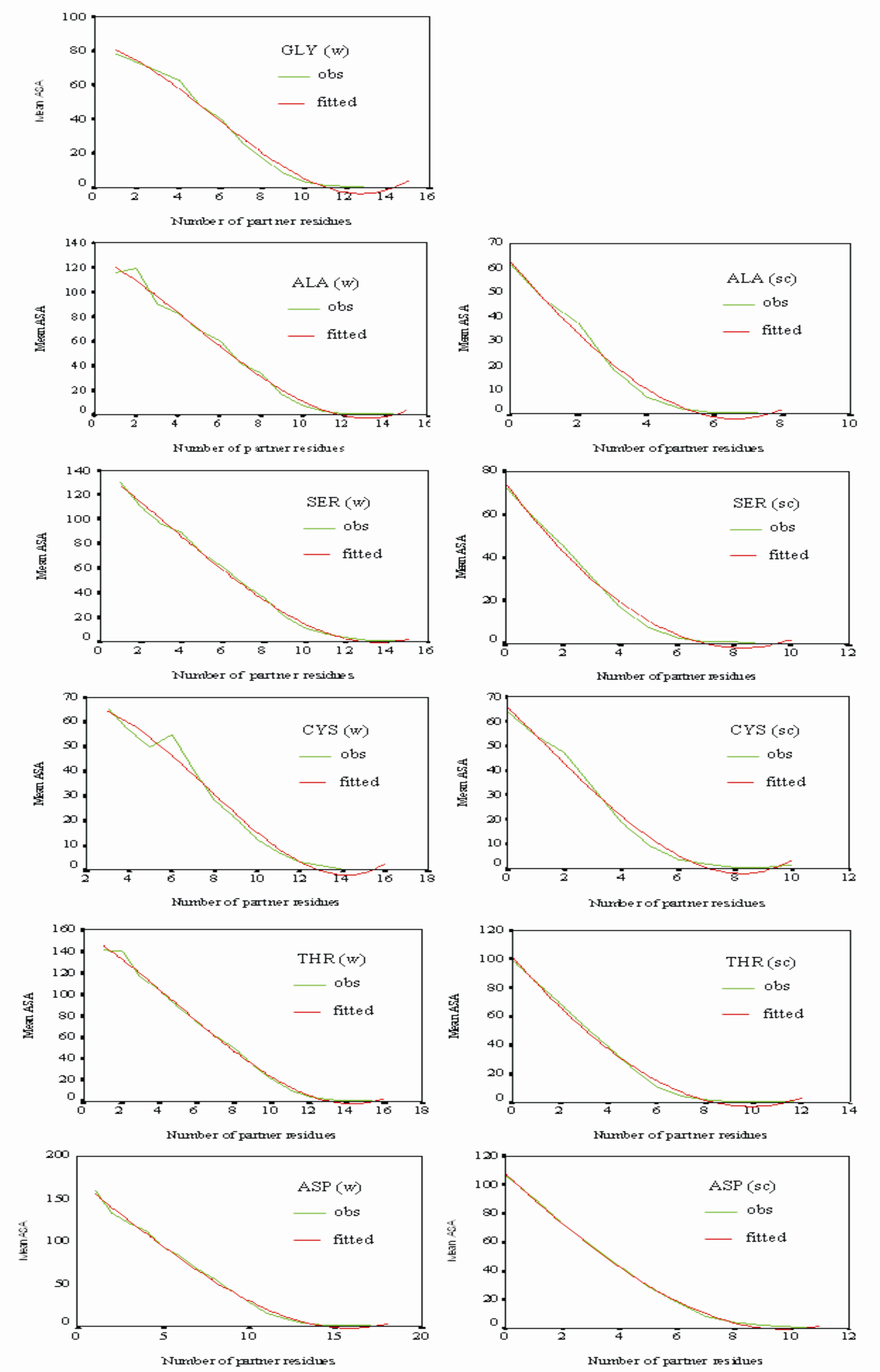


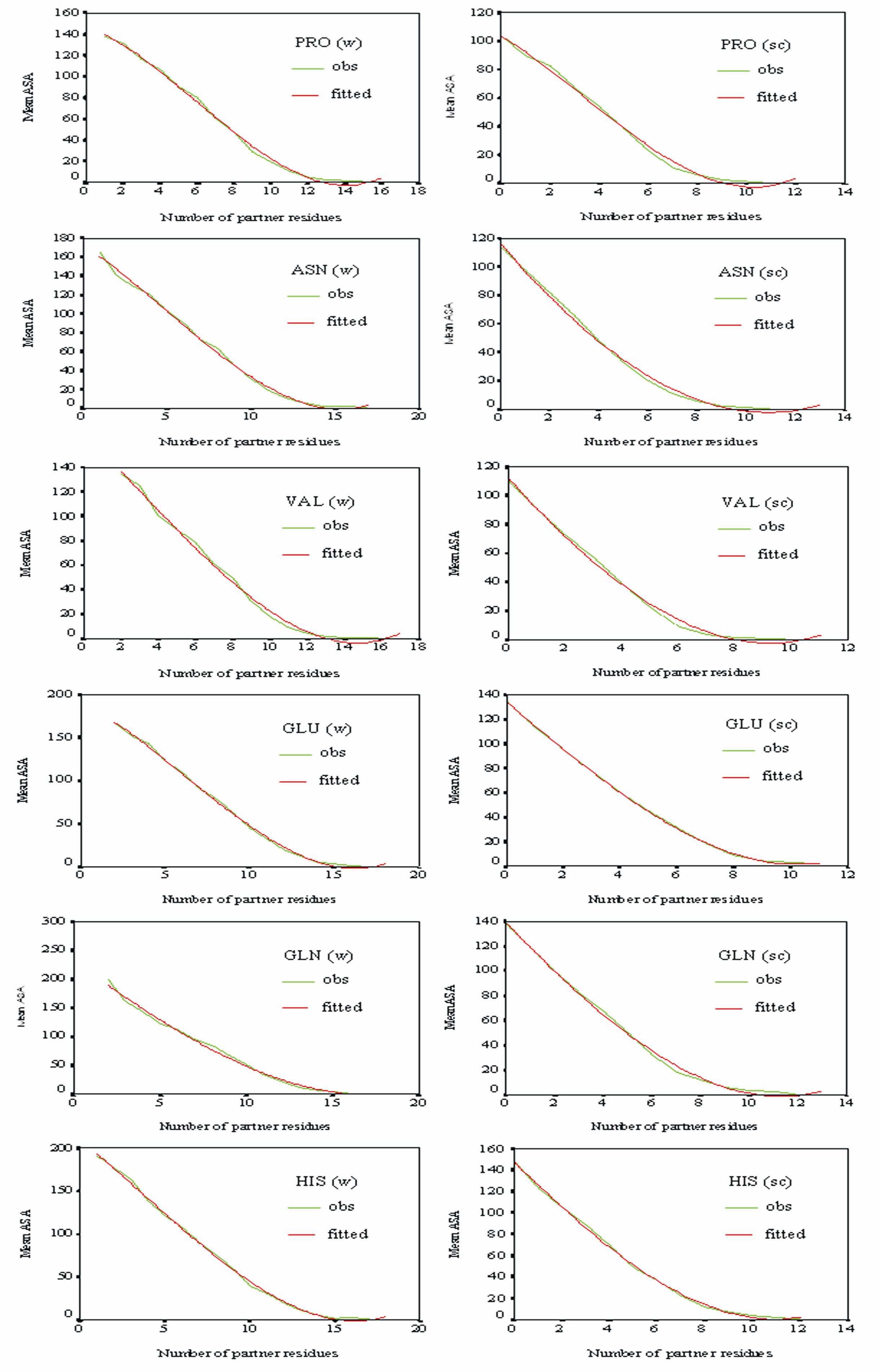


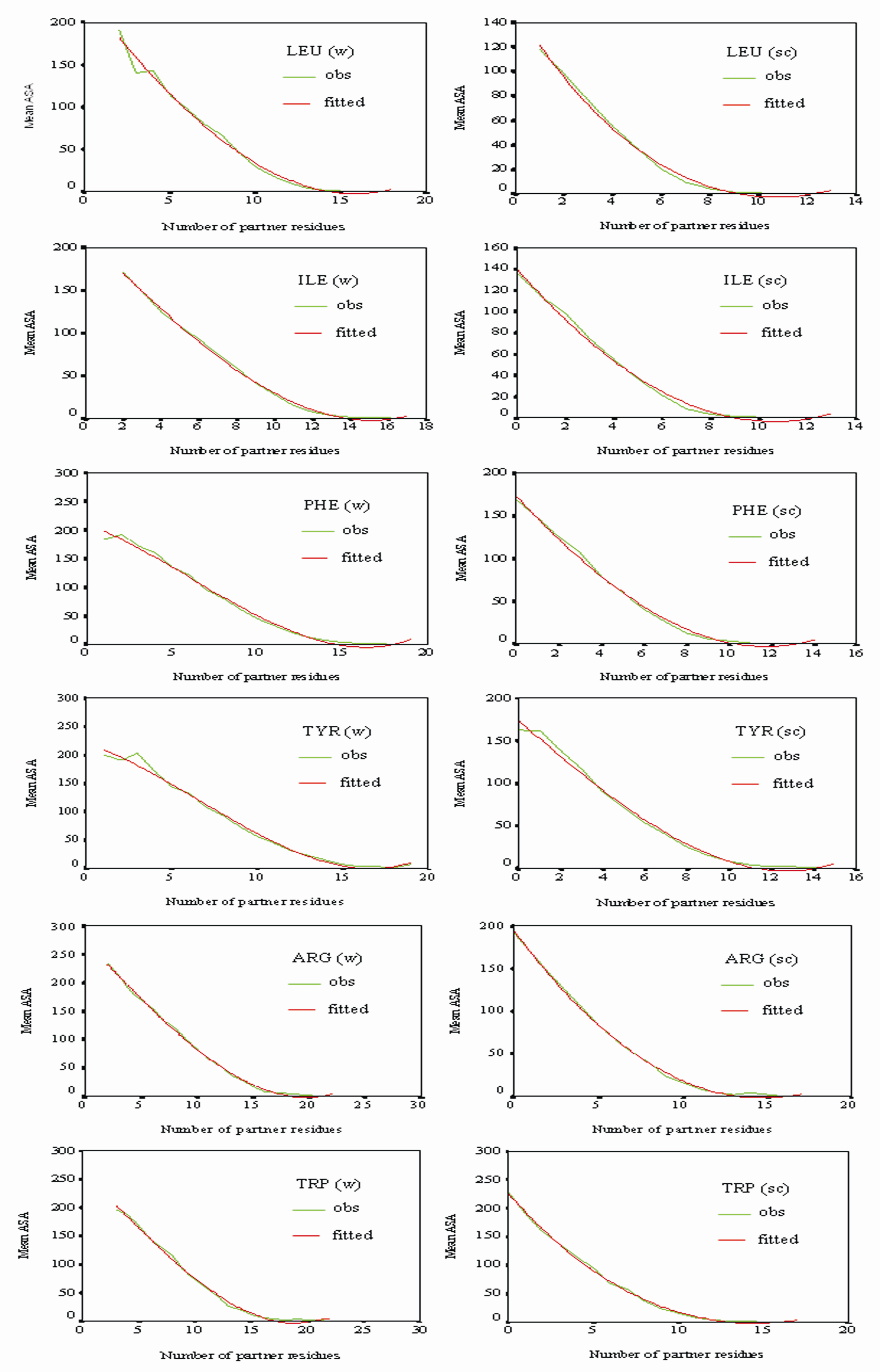


Figure 8. Plot of the difference, (ASA_calc_ – ASA_obs_), for 275 individual PDB files. The residue-based ASA_calc_ is the calculated ASA, obtained by summing up the values for all the residues in the structure; the latter are derived from their PNs using equations given in Table 1. The atom-based ASA_calc_ is obtained using exponential equations and atom-based PNs given in [7]. ASA_obs_ is the observed ASA for the structure, as given by the program NACCESS.


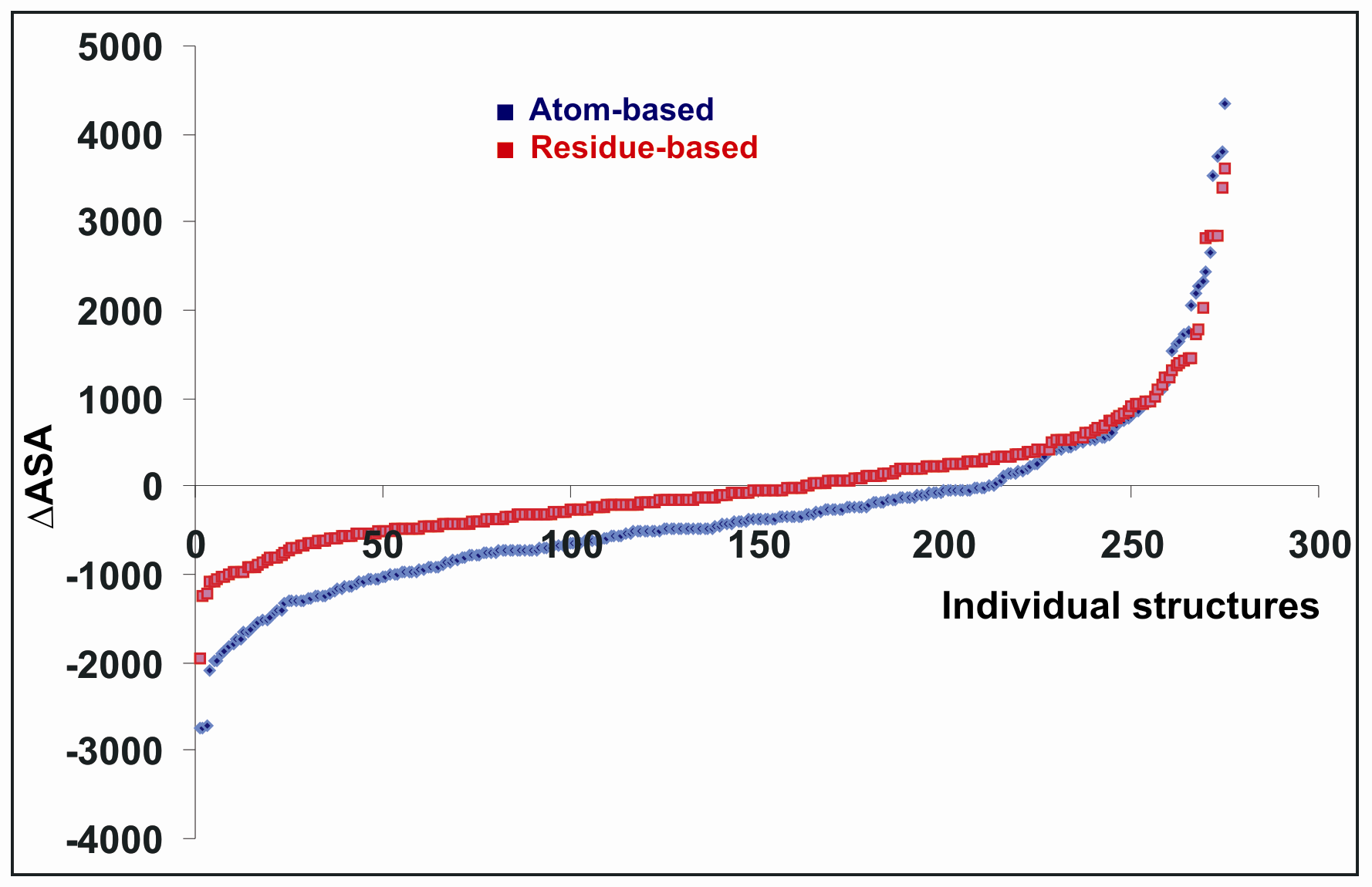

Supplement: Additional File 1 — Supplementary data. Table S1. Observed and calculated ASAs, and the match between them, in different protein structures. Figure S1. Variation of the mean accessible surface area (Å2) with partner number for residues other than Lys and Met. Figure S2. Plot of the difference, (ASAcalc – ASAobs), for 275 individual PDB files. [file 1471-2105-10-103-S1.doc]
